# Whole Exome Sequencing Reveals Promising Genes Associated with Congenital Renal Parenchymal Anomalies in Greek Children

**DOI:** 10.3390/children13060752

**Published:** 2026-05-28

**Authors:** Anna Zisi, Charilaos Kostoulas, Athanasia Sesse, Chrysoula Kosmeri, Anastasios Serbis, Hane Lee, Ioannis Georgiou, Ekaterini Siomou

**Affiliations:** 1 Department of Pediatrics, University Hospital of Ioannina, 45500 Ioannina, Greece; zisi_anna@hotmail.com (A.Z.); aserbis@uoi.gr (A.S.); eksiomou@uoi.gr (E.S.); 2 Laboratory of Medical Genetics in Clinical Practice, Faculty of Medicine, University of Ioannina, 45110 Ioannina, Greece; chkost@uoi.gr (C.K.); a.sesse@uoi.gr (A.S.); igeorgio@uoi.gr (I.G.); 3 Child Health Department, Faculty of Medicine, University of Ioannina, 45110 Ioannina, Greece; 4 3billion Inc., Seoul 06160, Republic of Korea; hlee@3billion.io

**Keywords:** CAKUT, children, variants, genes, whole exome sequencing

## Abstract

**Highlights:**

**What are the main findings?**

- Whole exome sequencing is a valuable diagnostic tool for patients with CAKUT.
- Possible candidate genes involved in nephrogenesis were identified (BBS1, PKHD1, XPNPEP3, and KCTD1), along with a novel previously unreported variant in GREB1L: a gene associated with CAKUT.

**What are the implications of the main findings?**

- These results highlight additional genes that could be involved in CAKUT.
- The findings may provide further insight into the complex genetic architecture of CAKUT.

**Abstract:**

Background: Congenital anomalies of the kidney and urinary tract (CAKUT) comprise a broad spectrum of malformations and constitute the leading cause of end-stage kidney disease (ESKD) in childhood. Despite extensive research, a monogenic cause is identified in only ~10% of cases, while variable penetrance and expressivity suggest a more complex disease mechanism. Epigenetic and environmental factors have also been implicated, further complicating efforts to elucidate the etiology of these anomalies. Methods: Whole exome sequencing (WES) was performed in 47 individuals with isolated, non-syndromic congenital renal parenchymal anomalies. Results: Variants in four genes (*BBS1*, *PKHD1*, *XPNPEP3*, and *KCTD1*) were identified, each of which has an established role in nephrogenesis and is implicated in syndromic disorders in which CAKUT can occur as part of the clinical spectrum. In addition, a variant in *GREB1L* was detected, a gene previously associated with CAKUT. The WES analysis identified candidate variants in 10.6% of patients, consistent with diagnostic yields reported in comparable CAKUT studies. The genes harboring variants are involved in key biological processes, including signaling pathways, ciliary function, and mitochondrial biology, supporting their relevance for further investigation. Conclusions: Our findings support WES as a valuable tool for identifying clinically relevant variants and expanding the genetic landscape of CAKUT.

## 1. Introduction

Congenital anomalies of the kidney and urinary tract (CAKUT) represent a heterogeneous group of structural defects that arise during renal and urinary tract development. These malformations account for approximately 20–30% of congenital abnormalities detected by prenatal ultrasound and occur in approximately 3–6 per 1000 live births, making CAKUT one of the most common causes of pediatric chronic kidney disease (CKD) and end-stage renal disease (ESRD) [1]. The CAKUT spectrum includes a wide range of renal parenchymal defects involving the renal parenchyma, such as renal agenesis, hypoplasia, dysplasia, and multicystic dysplastic kidney, as well as anomalies of the urinary tract, including ureteropelvic junction obstruction, vesicoureteral reflux, obstructive or refluxing megaureter, and urethral abnormalities such as posterior urethral valves [2].

Although many cases are sporadic, familial clustering occurs in up to 20%, indicating a strong genetic contribution [3]. Monogenic causes account for approximately 12–20% of CAKUT cases, with the highest diagnostic yield observed in syndromic forms, while 4–11% are linked to copy number variants. The genes *PAX2* and *HNF1B* are among the most frequently implicated, together explaining up to 15% of cases. Epigenetic mechanisms influencing nephron differentiation have also been implicated in CAKUT pathogenesis [4,5,6].

Recent developments in next-generation sequencing (NGS), particularly whole exome sequencing (WES), have improved the detection of rare pathogenic variants in genetically heterogeneous disorders. WES studies in CAKUT cohorts report diagnostic yields of 11–14%, enabling the discovery of novel disease genes [7].

Although CAKUT includes a wide range of manifestations and many cases may remain asymptomatic and not significantly affect quality of life, early diagnosis is essential for proper family counseling, as well as for timely intervention, even at the fetal stage [8]. The integration of molecular diagnostics into nephrology care pathways is expected to reduce diagnostic delays, healthcare costs, and clinical uncertainty.

The aim of this study was to investigate the genetic architecture of CAKUT in a pediatric cohort of Greek children with congenital renal parenchymal anomalies, using WES to evaluate the clinical significance of identified variants.

## 2. Materials and Methods

### 2.1. Patients

From April 2019 to March 2023, children with congenital renal parenchymal anomalies were prospectively enrolled. Patient history, physical examination findings, and laboratory data were recorded. The radiologic workup underlying the diagnosis of congenital anomalies of the renal parenchyma included abdominal ultrasound, voiding cystourethrography, technetium 99m dimercaptosuccinic acid (^99m^Tc-DMSA) scan and occasionally diuretic renogram. All the imaging studies were performed in the Radiological Department of the University Hospital of Ioannina. Inclusion criteria required evidence of isolated renal malformations with no known syndromic features, chromosomal imbalances, or extrarenal anomalies. Non-syndromic status was assessed through review of clinical history, physical examination, imaging findings, and available clinical records. Formal systematic evaluation by a clinical geneticist was not performed in all cases.

The study was performed in accordance with the Declaration of Helsinki, and written informed consent for genomic analysis was obtained from the legal guardians of all participants. The study protocol was approved by the Ethics Committee of our Hospital.

Among the renal parenchymal anomalies that were studied, unilateral renal agenesis (URA) was diagnosed by absence of one kidney on an abdominal ultrasound and ^99m^Tc-DMSA scan. Multicystic dysplastic kidney (MCDK) was diagnosed by multiple cysts on ultrasound, and absence of function on ^99m^Tc-DMSA. Since renal hypoplasia and dysplasia are histopathological diagnoses, we used the term renal hypodysplasia (RHD) for kidneys with reduced renal length and regular outline in addition to reduced corticomedullary differentiation. Although ductal or periglomerular cysts may be present, their absence does not exclude a diagnosis of dysplasia [9]. For diagnostic accuracy, only patients with severe RHD were included (split function ≤ 35%). Moreover, only patients with RHD and no anomalies in the ipsilateral lower urinary tract were enrolled.

### 2.2. Methods

#### 2.2.1. Genomic DNA Extraction and Sequencing

Genomic DNA was extracted from peripheral blood cells using the QIAsymphony DNeasy kit. The exome sequencing library was generated using the NEXTflex Rapid DNA-Seq kit 2.0 (Revvity, Waltham, MA, USA). Exome capture was performed using xGen Exome Research Panel v2 (Integrated DNA Technologies, Coralville, IA, USA) and sequencing was performed using NovaSeq 6000 (Illumina, San Diego, CA, USA) as 150 bp paired-end reads.

#### 2.2.2. Variant Calling and Annotation

Sequencing data was analyzed as previously described. Briefly, sequencing reads within the FASTQ files were uniquely aligned to the Genome Reference Consortium Human Build 37 (GRCh37) and Revised Cambridge Reference Sequence (rCRS) of the mitochondrial genome using BWA-MEM2 (v2.2.1) to generate BAM files [10]. Approximately 98~99% of the targeted bases were covered to a depth of ≥20×. Variants were called following the GATK best practices (GATK v4.2.14) for single-nucleotide variants (SNVs) and small insertions/deletions (INDELs) [11]. 3bCNV, an internally developed tool (manuscript in preparation), was used to call copy number variants (CNVs). The sensitivity and specificity of 3bCNV for calling heterozygous deletions and duplications that are larger than 30kb and spanning at least 3 consecutive coding exons, excluding NGS dead zones [12], were >99.9%. The analytical resolution was higher, reaching single-exon level, for homozygous or hemizygous deletions.

#### 2.2.3. Variant Prioritization and Interpretation

Initial variant annotation was performed using our internally designed bioinformatics pipeline, EVIDENCE v4 [13], which incorporates the Ensembl Variant Effect Predictor (VEP). The interpretation and prioritization followed a structured, sequential workflow. Variants were first excluded if they were too common in the population and defined as a minor allele frequency (MAF) >5% in the gnomAD database or >1% in our internal database (consistent with BA1/BS1 criteria), although well-established pathogenic and likely pathogenic variants were retained. Following frequency filtering, EVIDENCE v4 automatically classified variants based on a customized framework utilizing the foundational 2015 American College of Medical Genetics and Genomics (ACMG) guidelines [14,15,16,17], incorporating relevant internal adaptations. Subsequently, non-coding variants such as synonymous or intronic variants were filtered out unless they were predicted to impact splicing (SpliceAI score ≥ 0.2).

The retained variants were prioritized on clinical correlation, utilizing GEBRA, an internally developed SaaS platform that calculates phenotype similarity by integrating the patient’s specific Human Phenotype Ontology (HPO) terms to prioritize genes across the whole exome. To account for the inherent complexities of variant interpretation, particularly for criteria requiring contextual biomedical literature review, all automated classifications underwent a mandatory manual verification process in the context of the patient’s phenotype, relevant family history and previous test results provided by the ordering physician. Variants were evaluated under both autosomal dominant and recessive (including compound heterozygous) inheritance models. Because parental DNA samples were unavailable, strict segregation analysis could not be performed; thus, prioritization heavily weighed variant rarity and functional impact. Ultimately, only variants deemed clinically significant and relevant to the patient’s primary clinical indications at the time of variant interpretation were included in the reports.

## 3. Results

### 3.1. Cohort and Phenotypic Classification

A total of 47 unrelated Caucasian children (31 boys and 16 girls), diagnosed with isolated congenital anomalies of renal parenchyma, were included in the study. The mean age at the time of diagnosis was 24 months. Of these patients, 24 were referred to our department for evaluation and further work-up due to isolated abnormal prenatal ultrasound findings of the urinary tract. The remaining 23 patients were diagnosed postnatally, mainly following an episode of febrile urinary tract infection (10 patients) or during other incidental evaluations (13 patients).

All enrolled patients had non-syndromic CAKUT, while none had a documented family history of congenital renal anomalies. Patients were phenotypically categorized into unilateral renal agenesis (*n* = 20), horseshoe kidney (*n* = 8), MCDK (*n* = 9), RHD (*n* = 5), and ectopic kidney (*n* = 5).

### 3.2. Genetic Findings

Among the 47 pediatric patients analyzed, prioritized variants of potential interest were identified in five individuals (10.6%), involving the genes *BBS1*, *PKHD1*, *XPNPEP3*, *KCTD1*, and *GREB1L*. These findings should not be interpreted as definitive molecular diagnoses, particularly for heterozygous variants in genes classically associated with autosomal recessive disease. Table 1 summarizes key clinical and genetic features of the five individuals harboring prioritized variants, including the variant classification, type of renal anomaly, gene involved, zygosity, and protein-level consequences. Moreover, a diagnosis of Turner syndrome was established in a female presenting with a horseshoe kidney, which was confirmed by karyotype analysis, demonstrating a mosaic karyotype: mos 45X[30]/46XX[20]. This case was not included in the WES-based candidate variant detection rate, as the diagnosis was established by cytogenetic testing.

In the majority of patients, no pathogenic variants were identified. As previously mentioned, pathogenic, likely pathogenic, or variants of uncertain significance (VUS) were detected in only five individuals. Below, we describe in detail the identified variants, focusing on their genomic context, predicted impact, and association with CAKUT phenotypes in individual patients.

A *BBS1* variant c.1169T>G (p.Met390Arg) was identified in a female patient diagnosed with MCDK. *BBS1* has been implicated in Bardet–Biedl syndrome (BBS), a rare autosomal recessive disorder characterized by a pleiotropic phenotype. This missense variant is observed at an extremely low frequency in the gnomAD v2.1.1 database (total allele frequency: 0.00157%). Functional studies provide strong experimental evidence that this variant impairs protein function [18]. Computational predictions further support its deleterious impact, with a REVEL score of 0.66 and a 3Cnet score of 0.98. This variant has been classified in ClinVar as Pathogenic/Likely Pathogenic in 26 submissions. The *BBS1* variant identified in our patient was found in heterozygosity. Although this variant is classified as pathogenic according to ACMG/AMP criteria in the context of Bardet–Biedl syndrome, the presence of a single heterozygous variant is insufficient to establish a molecular diagnosis of this autosomal recessive disorder or to prove causality for the observed MCDK phenotype.

*PKHD1* harboring the c.5060T>C (p.Ile1687Thr) variant was found in a patient with MCDK. *PKHD1* is associated with autosomal recessive polycystic kidney disease (ARPKD), a condition characterized by enlarged, echogenic kidneys with fusiform dilatation of the collecting ducts. The substitution occurs in the fibrocystin extracellular domain, with damaging scores across SIFT, REVEL (0.95), and CADD (24.7), and is observed at an extremely low allele frequency (0.001%) in the gnomAD database. The variant in *PKHD1* was detected in heterozygosity and was classified as likely pathogenic according to ACMG/AMP criteria in the context of autosomal recessive polycystic kidney disease. However, no second pathogenic allele was identified, and therefore, this finding should be regarded as a monoallelic candidate variant of uncertain clinical contribution in this patient.

In the *XPNPEP3* gene, a frameshift variant (c.260_266del; p.Ala87GlyfsTer19) was detected in a male with renal hypodysplasia. Variants in *XPNPEP3* have been implicated in nephronophthisis, a tubulointerstitial nephropathy characterized by progressive renal function loss. This variant introduces a premature stop codon upstream of the catalytic AMP_N and prolidase domains, and is predicted to trigger nonsense-mediated decay (NMD), resulting in complete loss of protein function. Loss-of-function (LOF) is a well-established disease mechanism for *XPNPEP3*, with 19 previously reported pathogenic LOF variants. The affected exon contains four pathogenic variants, and the truncated region includes 20 known pathogenic mutations, underscoring its critical functional role. The variant was identified in heterozygosity in our patient. The *XPNPEP3* variant, reported here for the first time, is predicted to cause loss of function and warrants consideration as a potential contributor to the observed renal phenotype. Given its absence from gnomAD and predicted loss-of-function effect, the variant may fulfill criteria for likely pathogenicity in the context of autosomal recessive *XPNPEP3*-related nephropathy. However, because it was identified as a single heterozygous variant, its contribution to renal hypodysplasia in this patient remains uncertain and should be considered hypothesis-generating.

A previously unreported missense variant (c.7A>C; p.Asn3His) in the *GREB1L* gene, variants of which have been associated with CAKUT, was identified in a patient with unilateral kidney agenesis. The variant is located in exon 1, near the transcriptional start site. The variant was identified in the heterozygous state. This variant is absent from the gnomAD database, indicating it is extremely rare or potentially novel in the general population and has not been reported in ClinVar, nor has it been associated with CAKUT. Although classified as a VUS according to ACMG criteria, the known role of *GREB1L* in kidney development, together with the variant’s conservation and proximity to regulatory elements, supports its potential relevance. No additional validation testing was performed, as the variant call was of high quality.

Finally, a missense variant in the *KCTD1* gene, c.1910A>G (p.Asn637Ser), was identified in a female patient with MCDK. *KCTD1* is associated with autosomal dominant Scalp–Ear–Nipple (SEN) syndrome, a disorder characterized by distinctive scalp, ear, and nipple anomalies, while some affected individuals have also exhibited CAKUT phenotypes. *KCTD1* inhibits the activity of several transcription factors, thereby regulating critical gene expression programs during development. The p.Asn637Ser substitution affects a conserved residue within the BTB domain, potentially disrupting its interaction with transcriptional partners and altering transcriptional repression dynamics. Τhis missense variant identified in heterozygosity in *KCTD1*, is extremely rare in the general population (gnomAD allele frequency 0.001%) and in silico predictions (REVEL 0.32; 3Cnet 0.04) do not suggest a deleterious effect on protein function. Nevertheless, based on ACMG criteria, this variant was classified as likely pathogenic.

## 4. Discussion

This study presents a comprehensive WES analysis in a carefully phenotyped cohort of 47 unrelated pediatric patients presenting with non-syndromic congenital renal parenchymal anomalies. Potentially relevant pathogenic or likely pathogenic variants were detected in 10.6% of patients, which aligns with previously reported WES studies in CAKUT, where the diagnostic yield ranged between 4.8 and 14% [19], reinforcing the complex genetic architecture of renal tract malformations. Several identified variants were monoallelic findings in genes classically associated with autosomal recessive disorders (*BBS1*, *PKHD1*, and *XPNPEP3*). In the absence of segregation analysis, identification of a second pathogenic allele, or functional validation, these variants should be interpreted cautiously and considered candidate or hypothesis-generating findings rather than definitive molecular diagnoses. Their possible contribution to CAKUT may reflect genetic susceptibility, modifier effects, or incidental carrier status, particularly given the absence of parental segregation analysis. Finally, the relatively small cohort size in our study should be taken into consideration when interpreting genotype–phenotype associations.

Our findings align broadly with the international genetic model of CAKUT: while monogenic diagnosis is possible, it remains rare. Currently, over 54 genes have been implicated in CAKUT, accounting for roughly 12–20% of cases, and an additional 4–11% of cases are attributable to copy number variants (CNVs) [6]. The results of our study, with a CAKUT-relevant positive genetic finding rate of approximately 10.6%, align with the low-to-moderate diagnostic yields reported in similar international cohorts [20].

In the present study, variants were identified in the following genes: *BBS1*, *PKHD1*, *XPNPEP3*, *GREB1L*, and *KCTD1*. Additionally, one female patient with a horseshoe kidney was found to have Turner syndrome (45, XO).

### 4.1. BBS Gene Variant

A heterozygous *BBS1* variant was identified in a female patient with MCDK. Homozygous *BBS1* variants cause Bardet–Biedl syndrome (BBS), a rare autosomal recessive ciliopathy characterized by multisystem involvement. It is associated with variable renal anomalies, including renal agenesis, dysplasia, cysts, horseshoe kidney, and vesicoureteral reflux [21]. BBS is genetically heterogeneous, with at least 26 causative genes involved in primary cilium function and signaling pathways, including non-canonical Wnt signaling, which is important for nephrogenesis [22,23]. Although the role of *BBS* genes in isolated CAKUT remains unclear, increased CAKUT frequency has been reported in heterozygous relatives of BBS patients, raising the possibility that monoallelic variants could act as susceptibility factors in selected contexts; however, this remains unproven and may also represent an incidental finding [24]. Since only one *BBS1* variant was detected in our study, additional testing (e.g., Multiplex ligation-dependent probe amplification, MLPA) may help exclude a second undetected alteration. Nevertheless, the well-established role of BBS genes in nephrogenesis and the frequent renal involvement observed in patients with BBS support further investigation of these genes in CAKUT cohorts to better clarify their potential contribution to disease susceptibility.

### 4.2. PKHD1 Gene Variant

A heterozygous variant in the *PKHD1* gene was found in a patient from our study with MCDK. Variants in the *PKHD1* gene cause ARPKD, characterized by widespread renal cysts and congenital hepatic fibrosis. The encoded protein fibrocystin regulates planar cell polarity and tubular architecture [25,26]. Although carriers are generally asymptomatic, studies have reported medullary sponge kidney-like findings in heterozygous carriers and in mouse models [27,28]. In mice, *HNF1B* regulates *PKHD1* transcription, and its disruption leads to renal cyst formation, suggesting a possible role for *PKHD1* in CAKUT pathogenesis [29]. Given the key role of *HNF1B* in human CAKUT, this interaction supports a potential role for *PKHD1* in CAKUT pathogenesis. In our cohort, the heterozygous *PKHD1* variant identified in a patient with MCDK may represent a candidate susceptibility finding; however, its clinical contribution remains uncertain in the absence of a second pathogenic allele or functional evidence.

### 4.3. XPNPEP3 Gene Variant

A novel heterozygous truncating variant in *XPNPEP3* was detected in a patient with renal hypodysplasia. *XPNPEP3* encodes a mitochondrial aminopeptidase involved in protein maturation and ciliary function. Loss-of-function variants cause an autosomal recessive nephronophthisis-like nephropathy characterized by renal cysts and progressive kidney failure [30]. Although several developmental signaling pathways have been associated with nephronophthisis (NPHP), the molecular basis and pathogenesis remain unclear. A full understanding of *XPNPEP3*, particularly its mitochondrial role and its impact on renal ciliary function, is currently incomplete [31]. At present, there is insufficient evidence to directly associate heterozygous *XPNPEP3* variants with CAKUT phenotypes such as renal hypodysplasia. Additional functional and genetic studies are required to clarify the role of this gene in nephrogenesis.

### 4.4. GREB1L Gene Variant

A heterozygous variant in *GREB1L* was identified in a patient with unilateral renal agenesis. *GREB1L* participates in retinoic acid signaling, a pathway essential for kidney development and ureteric bud formation [32,33]. Variants in this gene have been reported in patients with renal agenesis, hypodysplasia, hydronephrosis, and other CAKUT phenotypes, although genotype–phenotype correlations remain variable [34,35]. The identified *GREB1L* variant is novel and currently classified as a VUS. Given the established association of *GREB1L* with renal developmental anomalies, this finding may be relevant to the patient’s unilateral renal agenesis; however, its pathogenic significance remains uncertain pending segregation studies and functional validation.

### 4.5. KCTD1 Gene Variant

A variant in *KCTD1* was found in a patient with MCDK. Pathogenic variants in *KCTD1* cause Scalp–Ear–Nipple (SEN) syndrome and affect WNT/β-catenin signaling, a key pathway in kidney development [36,37,38]. *KCTD1* also functions downstream of AP-2β and contributes to distal convoluted tubule differentiation [39]. Although KCTD1 participates in developmental signaling pathways relevant to nephrogenesis, direct associations with isolated CAKUT remain limited. Therefore, the clinical significance of the identified variant remains uncertain and should currently be regarded as a candidate finding of research interest.

One patient with a horseshoe kidney was diagnosed with Turner syndrome. This case was not included in the WES-based candidate variant detection rate, as the diagnosis was identified through karyotype testing. Turner syndrome occurs in 1 to 2500–3000 female births and is associated with renal anomalies in up to 70% of cases, most commonly a horseshoe kidney, but also hydronephrosis, ureteral stenosis, duplex collecting system, solitary kidney, MCDK, and renal cysts [40]. According to studies, it is recommended that all individuals with Turner syndrome undergo routine nephrological screening upon diagnosis. Therefore, early identification and ongoing monitoring are essential for the effective management of potential renal complications [41].

Interpretation of genetic variants in patients with CAKUT typically focuses on genes with well-established pathogenic roles, such as those included in curated diagnostic panels (e.g., ClinGen and PanelApp). In the present study, the use of WES enabled the analysis of all protein-coding regions of the genome, allowing for a broader and less hypothesis-driven search for potentially pathogenic variants.

### 4.6. Limitations of the Study

Potentially relevant variants were detected in 10.6% of patients. A possible genetic etiology remained unresolved in 89.4% of the cohort. This underscores key limitations of WES, particularly its limited ability to detect deep intronic variants, structural rearrangements (including copy-number variants), and epigenetic modifiers.

Interpretation of these findings is limited by the absence of parental segregation analysis and functional validation. The lack of segregation analysis in our patients also represents a limitation of this study, as it may have enabled a more precise and clinically informative interpretation of the identified variants. In particular, the absence of parental testing complicated the interpretation of VUS. Therefore, the identified variants should be considered candidate variants, and their contribution to the phenotype cannot be definitively established.

Moreover, the likely contribution of oligogenic and polygenic interactions to CAKUT, supported by recent genome-wide and burden analyses, highlights the need for extended analytical frameworks, including genome sequencing, transcriptomics, and epigenomic profiling.

## 5. Conclusions

Our findings suggest that WES can be a useful exploratory tool for studying kidney tissue abnormalities in children. Candidate variants were found in 10.6% of patients, but these results should be interpreted with caution because segregation and functional studies were not available. Single-copy variants in recessive genes may act as contributing factors or incidental findings rather than the direct cause of disease. Mono-allelic variants in genes classically associated with recessive inheritance (*BBS1* and *PKHD1*), as well as variants in candidate regulatory genes (*KCTD1* and *GREB1L*), should be interpreted with caution and may represent potential disease modifiers or contributory factors rather than definitive causal variants. Overall, WES highlights both established and emerging genetic pathways in CAKUT and supports improved diagnosis and personalized management in pediatric nephrology.

## Figures and Tables

**Table 1:** Genetic variants identified via exome sequencing in patients with congenital renal parenchymal malformations.

Sex	Phenotype	Gene	Associated Disease (OMIM)	Zygosity	Transcript	rsID	Variant	Protein Change	ACMG Classification	ACMG Criteria
F	MCDK	*BBS1*	BBS (209900)	Heterozygous	NM_024649.5:c.1169T>G	rs113624356	c.1169T>G	p.Met390Arg	Pathogenic	PS1_S, PS3_S, PM2_M, PP3_P
M	MCDK	*PKHD1*	ARPKD (263200)	Heterozygous	NM_138694.4:c.5060T>C	rs794727566	c.5060T>C	p.Ile1687Thr	Likely Pathogenic	PS1_S, PM2_M, PPS_P
M	RHD	*XPNPEP3*	NPHPL1 (613159)	Heterozygous	NM_022098.4:c.260_266del	NA	c.260_266del	p.Ala87GlyfsTer19	Likely Pathogenic	PVS1_VS, PM2_M
M	URA	*GREB1L*	RHD (617805)	Heterozygous	NM_001142966.3:c.7A>C	NA	c.7A>C	p.Asn3His	VUS	PM2_M, PP2_P
F	MCDK	*KCTD1*	SEN (181270)	Heterozygous	NM_001142730.3:c.1910A>G	rs767300513	c.1910A>G	p.Asn637Ser	Likely Pathogenic	PS1_P, PS2_S, PM2_M, PP2_P

ARPKD: Autosomal Recessive Polycystic Kidney Disease, M: Male; F: Female; RHD: Renal Hypodysplasia; URA: Unilateral Renal Agenesis; NPHPL1: Nephronophthisis-Like Nephropathy 1; SEN: Scalp–Ear–Nipple Syndrome; NA: Not Applicable; ACMG: American College of Medical Genetics and Genomics; VUS: Variant of Unknown Significance.

## Data Availability

The datasets generated during and/or analyzed during the current study are available from the corresponding author on reasonable request.

## Abbreviations

The following abbreviations are used in this manuscript:

CAKUT	Congenital anomalies of the kidney and urinary tract
CKD	Chronic kidney disease
WES	Whole exome sequencing
ESKD	End-stage kidney disease
RHD	Renal hypodysplasia
URA	Unilateral renal agenesis
